# Nature, Nurture and Evolution of Intra-Species Variation in Mosquito Arbovirus Transmission Competence

**DOI:** 10.3390/ijerph10010249

**Published:** 2013-01-11

**Authors:** Walter J. Tabachnick

**Affiliations:** Florida Medical Entomology Laboratory, University of Florida, IFAS, 200 9th St. SE, Vero Beach, FL 32962, USA; E-Mail: wjt@ufl.edu; Tel.: +1-772-778-7200; Fax: +1-772-778-7205

**Keywords:** mosquitoes, arboviruses, vector competence, environmental determinants, genetic determinants, evolution

## Abstract

Mosquitoes vary in their competence or ability to transmit arthropod-borne viruses (arboviruses). Many arboviruses cause disease in humans and animals. Identifying the environmental and genetic causes of variation in mosquito competence for arboviruses is one of the great challenges in public health. Progress identifying genetic (nature) and environmental (nurture) factors influencing mosquito competence for arboviruses is reviewed. There is great complexity in the various traits that comprise mosquito competence. The complex interactions between environmental and genetic factors controlling these traits and the factors shaping variation in Nature are largely unknown. The norms of reaction of specific genes influencing competence, their distributions in natural populations and the effects of genetic polymorphism on phenotypic variation need to be determined. Mechanisms influencing competence are not likely due to natural selection because of the direct effects of the arbovirus on mosquito fitness. More likely the traits for mosquito competence for arboviruses are the effects of adaptations for other functions of these competence mechanisms. Determining these other functions is essential to understand the evolution and distributions of competence for arboviruses. This information is needed to assess risk from mosquito-borne disease, predict new mosquito-arbovirus systems, and provide novel strategies to mitigate mosquito-borne arbovirus transmission.

## 1. Introduction

One of the great moments in medical science occurred on August 27, 1900 when James Carroll of the U.S. Army Medical Corps stationed in Cuba allowed Jesse Lazear to place a potentially infectious *Aedes aegypti *mosquito on Carroll’s arm and Carroll allowed it to take a blood meal. This was believed to have been the cause of Carroll’s subsequent bout with yellow fever on August 29 from which he survived. These events developed into the series of experiments by Walter Reed, Carroll, Lazear and Aristides Agramonte demonstrating that yellow fever virus (YFV) was transmitted by mosquitoes. Unfortunately on September 25, 1900 Lazear lost his life to yellow fever before the conclusive experiments were completed. Our knowledge about mosquito transmission of pathogens has made enormous strides in the ensuing 100+ years with information about different species of mosquitoes, the pathogens that they transmit, their ecologies, their roles in causing human and animal diseases, and a variety of tools that can be used to control or mitigate their impact on human and animal health and well-being. Information about the factors which influence a mosquito’s ability to become infected and to transmit a particular pathogen has continued to grow. It is now well accepted that not every *Ae. aegypti* is equal in its ability to transmit YFV and that populations of *Ae. aegypti* show differences in their vector ability for YFV. It is ironic to consider it fortunate to maintain the U.S. Army’s research group’s resolve to pursue mosquito transmission that Carroll did get yellow fever and that the individual mosquito chosen for the initial infection of James Carroll was one of those *Ae. aegypti* competent for YFV transmission. 

The suite of factors that allow an arthropod that has encountered a pathogen to become infected and to transmit a particular pathogen once it encounters a susceptible host is defined as the arthropod’s vector competence for that pathogen. One can only admire the exquisite series of events that occur in a mosquito that result in vector competence and the transmission of a pathogen. The events comprising arthropod vector competence have been described elsewhere [1,2,3,4,5,6,7,8,9]. The process of vector infection begins when the pathogen enters the mosquito within a blood meal containing sufficient numbers of the pathogen to ensure some will encounter the epithelium where the blood has been deposited in the arthropod’s midgut. The pathogen must be able to cross the epithelium that has been termed the midgut infection barrier (MIB). Once in the epithelium the pathogen must replicate, cross the epithelium and escape the midgut into the hemocoel in a process termed the midgut escape barrier (MEB). The pathogen then must replicate in various mosquito tissues but ultimately some sufficient quantity of the pathogen must invade the mosquito’s salivary glands in a process overcoming the salivary gland infection barrier (SIB). There the pathogen replicates and ultimately must escape the salivary gland in the process described as the salivary gland escape barrier (SEB) upon subsequent blood feeding when it is injected into a susceptible animal host to complete the transmission cycle. This entire process can take several days to complete in the mosquito during a period called the extrinsic incubation period (EIP). Along the way there are a myriad of other arthropod factors in addition to the various barriers to the pathogen that may also influence the pathogen and the arthropod’s vector competence. The pathogen encounters arthropod digestive enzymes and digestive processes, intracellular processes and the arthropod’s immune system to name just a few processes that also influence vector competence.

It is widely appreciated that arthropod genetic factors and the environment both influence vector competence. However the complexity of how these factors interact with one another to determine variation in vector competence within a vector species is only beginning to be appreciated. The goal of this chapter is to explore this complexity with particular attention to the challenges in understanding determinants of vector competence, how this knowledge is essential in understanding and controlling vector-borne diseases, and to provide a perspective on the gaps in our understanding. This chapter focuses on mosquito competence for arthropod-borne viruses, the so called arboviruses. Extraordinary advances have occurred with other arthropods and the pathogens they transmit, particularly the *Anopheles* vectors of malaria, and these are discussed elsewhere [4,10,11,12,13,14,15,16]. The issues and perspective on mosquito competence for arboviruses reviewed here are also applicable to other arthropod vectors, the pathogens they transmit and other arthropod traits that contribute to arthropod transmission. The reader will be directed to a few select examples of mosquito competence for arboviruses to illustrate different concepts with apologies to the many other excellent studies that could not be included in the interest of available space for this brief review.

## 2. The Vector Competence Phenotype

The brief summary of the suite of factors described above as the different traits comprising vector competence is only a partial picture. Each of the traits included in this description can be thought of as a specific phenotype of the vector. Individual mosquitoes have a specific MIB, MEB, SIB, SEB, immune pathways, digestive processes, *etc*., that are the phenotypes for a specific pathogen. Many studies have addressed factors that influence each such phenotype. However, most studies have focused on the mosquito’s susceptibility to infection as the phenotype while there are a few studies on the ability of the infected mosquito to transmit the pathogen as another phenotype. All vector competence studies share the problem that the phenotypes being studied are generally not specifically defined. Generally each of the observed phenotypes actually encompasses several different mechanisms and there may be a variety of mechanisms that can result in the same phenotype. For example, the MIB phenotype likely consists of several different mechanisms, *i.e*., failure of the virus to attach to a cell receptor, digestive or immune processes that reduce viral reproduction or failure of the virus to replicate within the midgut epithelium. Each of these is a more specifically defined phenotype and each is a part of the MIB phenotype and a part of vector competence. The phenotype of vector susceptibility to infection includes the MIB and MEB processes, in addition to processes involved in immunity, digestion, viral replication, intracellular processes and all the mechanisms influencing each of these. The phenotype of transmission of the pathogen to an animal host consists of many different traits. It is a general phenotype since it includes all the processes and mechanisms in the vector that are required for transmission including all of the barriers and processes that one might encounter.

## 3. Determinants of Vector Competence

Although there are many studies characterizing variation in several of the broad components of vector competence, there has been little information to establish the primary causes for observed variability in vector competence within any vector species. In general the primary focus of most studies has been on laboratory based characterizations of vector susceptibility to infection. There have been far fewer studies on the dissemination of the pathogen out of the vector midgut that must occur for transmission. There are even fewer studies of salivary gland infection and far less work on actual transmission to the animal host.

What is evident from all of these studies is the widespread phenotypic variation in components of vector competence between mosquito populations no matter how the phenotype is defined. This has been most evident in the large number of studies showing geographic variation in the susceptibility to infection with various arboviruses between mosquito populations in various mosquito species. Phenotypic variation in susceptibility to arbovirus infection between populations of mosquitoes within a species has been found in every mosquito-arbovirus system studied for multiple populations [17,18,19,20,21,22,23,24,25,26,27,28,29,30,31,32,33,34,35,36,37]. There has been little similar work on population variation in mosquito ability to transmit an arbovirus due to the greater effort involved in characterizing the transmission phenotype in individual mosquitoes. Compared to simply measuring virus in a mosquito’s body to establish whether the mosquito is infected, it is more difficult and labor intensive to collect and detect virus in mosquito saliva that necessarily involves expending considerable yet wasted effort testing mosquitoes that may not even be infected or lack a disseminated infection. Phenotypic variation in the ability for an arbovirus to escape from the midgut and disseminate to other body tissues has also been observed [39,40,41,42]. There is evidence that phenotypic variation for infection, dissemination and transmission of West Nile virus (WNV) were independent phenotypes from one another in a population of *Culex pipiens quinquefasciatus* indicating that these phenotypes are controlled by different mechanisms [43]. 

The causes of variation resulting in the different competence phenotypes between individual mosquitoes within a vector species is a daunting challenge that has hardly been explored. The large number of studies showing phenotypic variation in competence for arboviruses between mosquito populations is strong evidence for genetic causes for this variation since each such study was conducted under laboratory conditions where each population tested in the particular study was likely exposed to similar environmental conditions. Hence since there is likely little environmental variation in the laboratory test within a particular study, the observed variation between populations is more likely the result of genetic factors. However despite this indirect evidence for genetic causes it is generally appreciated that there are likely genetic and environmental factors that contribute to the observed phenotypic variation in the laboratory and that both likely contribute to actual variation in Nature. Further, it is sobering to realize that laboratory tests of vector competence do not likely reflect naturally occurring phenotypic variation in either the mosquito and virus populations. Such studies cannot include the range of genetic variation available in Nature, and laboratory studies are unable to employ the range of environmental variability that occurs in Nature in the laboratory environment. 

## 4. Mosquito Genetic Factors Influence Mosquito Competence for Arboviruses

There is widespread variation within mosquito vector species for the different components of vector competence for arboviruses. There have also been many studies on the population genetics of mosquitoes using molecular markers that shows that there is a great deal of genetic differentiation that exists between mosquito populations and that there is genetic variation in general that exists within mosquito vector species [44,45,46,47]. For example, population genetic variation has been explored in the arbovirus vectors *Ae. aegypti* [32,48,49,50,51,52,53,54,55,56,57], *Ae. albopictus* [58,59,60,61,62], *Culex pipiens* complex mosquitoes [63,64,65,66,67,68], to name just a few. 

Although obtaining information about specific genetic factors that contribute to naturally occurring variation in competence for arboviruses within mosquito species has been difficult, substantial progress has been made since the subject was reviewed over the last 20 years [4,44]. There is hope that the application of mosquito genomics approaches to mosquito competence for arboviruses will result in greater progress in identifying more genetic factors [69,70]. There are only a few studies that have explored genetic variation in mosquito competence and even fewer that have sought to identify specific genes controlling mosquito variation in vector competence. Initial genetic studies showed that there was a genetic component to mosquito vector competence either through observing a response to selection for resistance and/or susceptibility to arbovirus infection and/or employing family studies showing the familial basis of the phenotype [23,71,72,73,74,75,76]. One of the few studies identifying a single genetic locus with a proposed genetic mechanism influencing insect susceptibility to infection with an arbovirus was in the biting midge, *Culicoides sonorensis*, and it’s susceptibility to infection with bluetongue virus (BTV) [77]. This rare demonstration showing a single locus that influenced susceptibility involved a mechanism of maternal inheritance and paternal imprinting. This provided an example of the potential genetic complexity that might occur in other vector-pathogen systems. Quantitative inheritance approaches have identified several genetic factors in the form of quantitative trait loci (QTL) that influence *Ae. aegypti* variation in vector competence for dengue virus (DENV) [78]. These studies identified regions of the *Ae. aegypti* chromosome that contained one or more genes that influenced variation in the midgut escape barrier for DENV [79,80,81]. The identified QTL among the seven QTL detected in these studies varied in their influence depending on the geographic origin and maintenance in the laboratory of the *Ae. aegypti *populations that were employed in the analysis though the specific identity or functions of these QTL have not been determined. These observations show the potential for great complexity in the genetic mechanisms influencing competence. This complexity is illustrated by the observation that different populations of *Ae. aegypti* contain different genetic factors and potentially different genetic mechanisms that influence variation in susceptibility. There are different QTLs in different populations that reside in different chromosomal regions that influence *Ae*. *aegypti* competence for DENV. Hence there is no reason to believe that competence variation in different populations of the same species is due to the same genetic mechanisms. For example, refractoriness for YFV in Asian *Ae. aegypti* may be due to completely different mechanisms than those producing refractoriness in *Ae aegypti* populations in the Caribbean. How many such mechanisms exist in a species such as *Ae. aegypti*? One approach to answering this question would be to perform simple complement tests [82] between diverse lines homozygous for particular phenotypes. If the same loci control a phenotype like susceptibility to infection the F1 offspring resulting from a cross between a resistant male from one line and a resistant female from another should also be resistant. If they are not then one is dealing with different genes or different complementation groups. The range of diverse complementation groups for any vector competence phenotype is unknown. The diversity of mechanisms in different mosquito populations is completely unknown.

A variety of genomic approaches have identified 1,000s of candidate genes that influence vector competence. For example, studies have involved proteomics and transcriptome profiling to identify genes involved in mosquito responses to arbovirus infection [83,84,85,86,87,88,89]. A genome-wide transcriptome profile for *Ae. aegypti* strains that differed in susceptibility to infection for DENV identified ca. 2,500 *Ae. aegypti* genes that had different responses when the two phenotypes were compared after challenge with DENV [87]. This large scale study provided independent support for many other previous studies by identifying many of the same genes. Among these were genes in the mosquito innate immune response pathways that have been shown to respond to infection with arboviruses like DENV [84,90,91,92,93,94,95]. When exposed to arboviruses mosquitoes respond with anti-microbial immune pathways like Janus kinase-signal transducer and activator of transcription (JAK/STAT) and Toll pathways [93,96], immune deficiency (IMD) [94,95] and RNA interference (RNAi) machinery [97,98,99,100,101]. Other genes previously implicated in *Ae. aegypti* vector competence and also identified in the genome-wide profile include genes controlling trypsin in the mosquito midgut [102] and genes controlling serine proteases [103] that may play a role in *Ae. aegypti* susceptibility to infection with DENV. Genes that have also been shown to play a role in mosquito competence include genes producing proteins that bind with an arbovirus [104,105,106,107,108,109,110,111,112,113]. A variety of *Ae. aegypti* midgut genes have been found to differ between resistant and susceptible strains of *Ae. aegypti* to DENV [114] and the importance of the midgut epithelium proteins has been observed in *Aedes taeniorhynchus* infected with Venezuelan equine encephalitis virus in the mosquito vector [115]. Though barriers to infection in the mosquito likely involve receptor proteins, conditions have been observed where differences in the competence of two strains of *Ae. aegypti* for DENV was not due to differences in the binding of DENV to midgut proteins [116]. Many diverse factors influence mosquito competence for arboviruses.

There is great complexity in the genetic factors that influence mosquito variation in vector competence for arboviruses. *Aedes aegypti* vector competence to DENV illustrates the involvement of suites of different genes in what has been described as gene networks [87] any of which might cause variations between individual mosquitoes and hence between populations of mosquitoes that could influence disease epidemiology. A variety of interacting intrinsic genetic factors influence *Ae. aegypti* competence for DENV [117]. The numbers of potential controlling genes that can influence the vector competence phenotype is impressive but this is not surprising considering the array of traits that comprise vector competence. It is a daunting challenge to consider that there is so little information about the extent of genetic variation in natural populations of mosquitoes for the genes that influence competence. As new genes are discovered it will be essential to determine which might be considered major genes with the greatest influence on the competence phenotype whether this is for the MIB, MEB, SIB, SEB, viral replication, immune pathways, *etc*. Then such genes will need to be characterized for polymorphisms and the extent of the effects of each polymorphism on phenotypic variation assessed. Ultimately it will be essential to characterize the population genetics of these genes, characterize their frequencies in different populations and then identify factors responsible for those frequencies. This information will be a first step in being able to understand how vector competence evolves and the role of vector competence in vector-borne disease epidemiology. It is expected this information will lead to novel approaches to modify vector populations to be of less danger. 

## 5. Environmental Factors Influence Mosquito Competence for Arboviruses

The challenge in understanding naturally occurring phenotypic variation in mosquito competence for arboviruses is compounded by the many studies describing the influence of different environmental factors on competence. Here anything that is non-genetic in origin in the mosquito that influences the mosquito competence phenotype will be considered as an environmental factor. Studies of environmental effects on competence for arboviruses generally have used the broadly defined phenotypes of susceptibility to arbovirus infection and less often the ability of infected mosquitoes to transmit. Since the identification of specific genes that have been shown to control any aspect of mosquito competence is still in its infancy, environmental influences on any specific genes have not yet been characterized. 

The most often studied environmental factor influencing mosquito competence for arboviruses is temperature. The temperature of a mosquito’s environment can influence a mosquito’s competence for an arbovirus in a number of different ways. For example as temperature increases virus replication will generally increase in a mosquito’s tissues. Many studies have provided support for the increase in virus replication in mosquitoes with increased temperature by observing increasing susceptibility to arbovirus infection in the laboratory at increasing temperatures and some have showed increased transmission as well [38,40,118,119,120,121,122,123,124,125,126,127,128,129,130,131,132]. Other studies have shown that variation in the external temperature can influence a mosquito’s ability to modulate replication of the virus in the mosquito’s cells [1,133]. The temperature at which the adult female mosquito is exposed during the EIP after the virus has entered the midgut through the acquisition of the blood meal influences the subsequent events involved in competence for the arbovirus. The impact of fluctuating temperatures during the EIP on vector competence have also been explored in an attempt to reflect what more likely occurs under natural conditions [132]. Other studies with a variety of mosquito-arbovirus systems have shown that temperature, nutrition and competition during the larval stage may also influence the subsequent vector competence for arboviruses of the resulting adult females [20,42,127,133,134,135,136,137,138]. The specific manner and which specific aspects of the suite of traits comprising vector competence are influenced by temperature are largely unknown. Some of the observed effects of temperature are due to increased viral replication at higher temperatures that often results in a shortening of the EIP. 

Though most studies of environmental influences on mosquito vector competence have involved the effects of temperature other studies have reported on the effects of other environmental factors. Exposure to insecticides in the adult or larval stages has been shown to influence mosquito competence for arboviruses [137,139,140], and so has humidity and the pH of the blood meal [1]. Different forms of the arbovirus, *i.e*., arbovirus serotypes or other genetic differences between viruses can influence a mosquito’s vector competence [25,41,141,142,143,144,145,146,147,148,149,150,151,152,153,154,155]. The amount of virus that a mosquito imbibes in the blood meal can influence mosquito competence generally with greater susceptibility to infection with an arbovirus when the mosquito is exposed to blood meals containing higher titers of the arbovirus [25,149,156,157,158,159]. Changes in the expression of genes involved in mosquito immunity that also interfere with *Ae. aegypti* competence for DENV have been observed due to infection with *Wolbachia* [160,161].

The large number of studies that demonstrate environmental effects on mosquito competence for arboviruses have hardly explored the potential complexity of environmental effects. Several studies have shown that environmental factors interact with one another in complex unpredictable ways to influence mosquito competence for arboviruses. For example, though increasing environmental temperature generally increases susceptibility to infection and may shorten the EIP needed for transmission, the effect of temperature on vector competence can be influenced by the virus dose in the blood meal and the age of the mosquito in *Cx. pipiens quinquefasciatus* exposed to WNV or St. Louis encephalitis virus (SLEV). The age of adult *Cx. pipiens quinquefasciatus* influenced their vector competence to SLEV but the influence of the mosquito’s age was dependent on both temperature and virus dose [162]. *Culex pipiens quinquefasciatus* exposed to WNV also showed interactions between different age classes but these effects were dependent on temperature and dose and the effects differed between two different colonies [163].These observations show the complexity of environmental factors and their effects on mosquito competence for arboviruses. The effects of environmental factors like external temperature, the age of the mosquito and virus dose as examples, were dependent on other factors in non-linear ways, and different populations or colonies of a species responded to the environment differently. The complexity of environmental influences was shown for *Cx. nigripalpus* competence for WNV where the effects on infection, dissemination and transmission were influenced differently by dose and the length of the EIP [159]. Further adding to the complexity is the observation that the observed interactions between environmental factors are dependent on unknown genetic factors in the mosquito that also show extensive variation. This was illustrated by different responses to the environment by different colonies of the same species [159]. The complexity of the genetic and environmental influences on vector competence might lead to suspicion that the resulting phenotype is a consequence of random factors. This is a misleading conclusion. The resulting phenotype is not random but it is the result of a host of contingent, inter-related factors. Unfortunately the great majority of the factors and their inter-relationships have not been identified.

**Figure 1:**
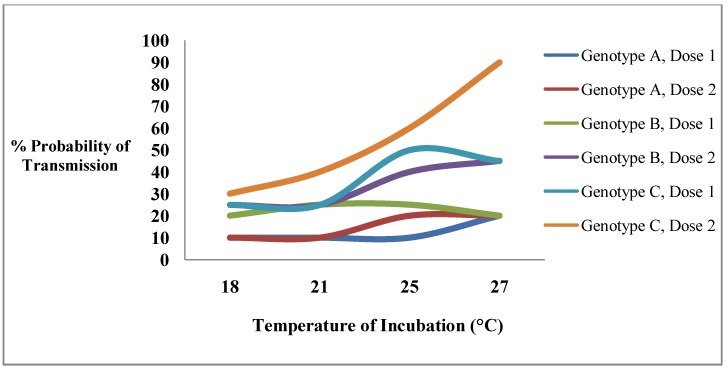
An example of the effects of two environmental factors (EIP, virus dose in the blood meal) and mosquito genotype on the probability of a mosquito transmitting an arbovirus. The norm of reaction of each genotype for temperature is dependent in a nonlinear way on the dose of the virus in the blood meal obtained by the mosquito.

The response of specific vector competence genotypes to the range of variation in any environmental factor, the norm of reaction of the genotype, has not been characterized and is likely very complex (Figure 1). For example, in this illustration the change in competence at different temperatures for genotype C is dependent on the dose of the virus. Therefore, since little is known about mosquito competence genotypes it should not be surprising that relationships between different environmental factors influencing vector competence have not been explored. There is no information about potential interactions nor is there any information in any mosquito-arbovirus system suggesting whether such relationships are linear in their effects on vector competence phenotypes or may have other shapes depending on the environment and genetic constitutions of the virus and mosquito. All observations to date have been made using laboratory studies employing a mosquito population or colony as a representative of naturally occurring variation in the mosquito species though actual information concerning the extent of genetic variation in any species of mosquito is not available. A similarly selected virus is usually employed in these studies to represent virus variation in Nature though the extent of virus variability in Nature is also uncertain. Finally the mosquito is exposed to the arbovirus in the laboratory under environmental conditions that likely represents only some selected portion of the range of environmental variation in Nature. It is no wonder that there is so little known about variation in competence and its causes. The observations that have been reported from these types of studies do not provide the ability to assess natural conditions and must be interpreted with great caution. As a result it is not possible to assess the vector competence of an entire species with assurance based on laboratory observations using a few selected mosquito populations.

## 6. The Importance of Vector Competence Variation in Arbovirus Epidemiology

**Figure 2:**
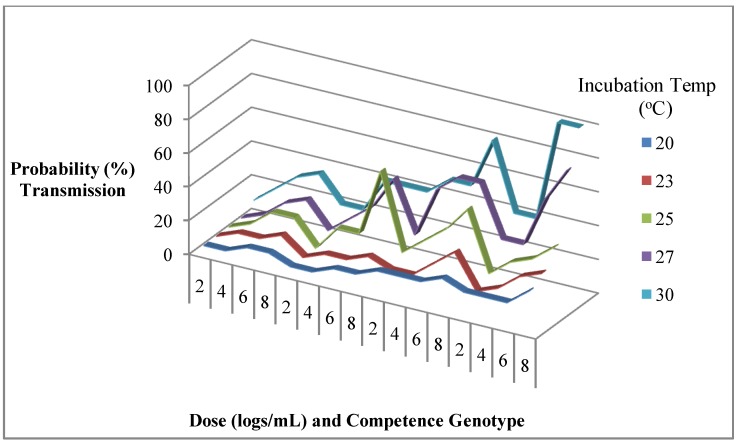
An example of transmission variation that is dependent on non-linear relationships between vector competence genotype, virus dose, and temperature of incubation. The epidemiologic significance of the variations in the probability of transmission such as shown here are unknown.

The evidence for extensive phenotypic variation in all aspects of mosquito vector competence is overwhelming. However the role that such intra-species variation contributes to arbovirus epidemiology has not been extensively explored. The observations of phenotypic variation illustrated above show that the observed variation was statistically significant within the particular study. Depending on the sample sizes used in a particular study, the magnitude of variation could be slight though statistically significant. Observing statistically significant differences within a study is not definitive proof that the observed variation is biologically meaningful with a measurable effect on mosquito-borne transmission and/or disease epidemiology. For example does a 10% statistically significant and therefore real difference in susceptibility to infection between two mosquito populations for an arbovirus translate into meaningful differences in pathogen transmission that also have an influence on disease epidemiology? Does 20% or 50% or 90% influence epidemiology? What does it mean for a population to have 70% of the mosquito population susceptible to infection, 70% of mosquitoes infected capable of dissemination out of the midgut and 60% of those with dissemination capable of transmission to a host animal? If the three traits are independent of one another then the probability of actual transmission for any mosquito in this population is ca. 30%. How does this compare with a population with 50% infection, 50% dissemination, 10% transmission and actual transmission probability then of 2.5% if the 3 traits are also independent of one another? Other things being equal does it mean that the population with 30% probability of transmission is more likely to support transmission than a population with 2.5% probability of transmission and that more mosquitoes would be needed to sustain the same level of pathogen transmission in the latter population? The epidemiologic significance of the hypothetical variation in transmission rates illustrated by Figure 2 is unknown.

This difference in transmission was observed when a relatively incompetent population of *Ae. aegypti* supported an epidemic of yellow fever in Nigeria [164]. Using a model [165] to estimate the number of bites needed to sustain YFV transmission, and observed infection rates of 30% in Caribbean *Ae. aegypti* compared to only 2% as the estimate of actual transmission in a Nigerian *Ae. aegypti* population, it was shown that ca. 15 times the biting intensity was needed to sustain YFV transmission in the Nigeria outbreak compared to what would have been needed with a more competent Caribbean *Ae. aegypti* population [164]. This yellow fever epidemic was likely due to the very high numbers of *Ae. aegypti* observed at the time in the regions of Nigeria with yellow fever. It was also believed that the low competence in the *Ae. aegypti* population in the region was a factor that prevented the epidemic from spreading to other regions [164]. 

Specific evidence linking observed variation in vector competence with the appearance of arboviral epidemics is lacking because there are many other factors that contribute to the appearance of an epidemic. Some studies have concluded that variation in pathogen epidemiology is likely caused by other factors influencing the mosquito-arbovirus system rather than variation in mosquito population competence for arboviruses. An often discussed epidemiological puzzle in mosquito-borne arbovirus epidemiology is the historical absence of yellow fever from Asia despite the presence of *Ae. aegypti* throughout Asia and the historic presence of DENV and dengue fever in Asia [166]. A transmission model suggested that the most likely explanation for the absence of yellow fever in Asia was the possibility of cross immunity between DENV and YFV, and the presence of both *Ae. albopictus* and *Ae. aegypti* in Asia for substantial DENV infection with cross protection against YFV [167]. YFV and DENV coexist in Africa and have occurred in the new World where there are very high ratios of *Ae. aegypti* compared to *Ae. albopictus* resulting in less dengue and less cross protection in these regions. It is interesting that the relatively lower competence for YFV by Asian *Ae. aegypti* [23] was not considered substantial enough to be the cause of YFV’s absence in Asia. An *Ae. aegypti* population with probability of infection of 20% did not substantially reduce the prevalence of YFV compared to mosquito populations with a probability of 70 and 80% according to the model [167]. The probability of infection represents only one component of competence. The potential for transmission by Asian *Ae. aegypti* in the natural environment is unknown. Therefore the low vector competence of Asian *Ae. aegypti* for YFV may yet be an important factor in the historic absence of YFV from Asia.

## 7. Challenges in Characterizing Causes of Variation in Mosquito Competence for Arboviruses

New technologies in gene sequencing, gene expression analyses, transcriptome profiles, and proteomics have greatly accelerated gene discovery in all fields of biology. Vector biology has benefitted as well as evidenced by the rapid progress in gene discovery that has identified hundreds of genes responding to mosquito competence infection with arboviruses. The rapid progress in gene discovery is the result of the technological advances in genomics. Richard Lewontin [168] pointed out that scientists are successful because they know it is important that they choose to pursue amenable problems using the best available technologies and resources. This has meant rapid progress in genomics and related issues because of the availability of new high throughput methods using automated sequencing, transcriptome profiling, PCR, and proteomics. The result however has been less attention to the more difficult biological problems that are not as amenable for study using the new technologies. These are the problems that require translating genomic information into understanding of function, form, phenotype, fitness effects and evolution. Despite the progress in gene discovery involved in mosquito competence shown in this review, we are only at the very beginning in meeting the challenge to understand translating genomics information into understanding of the form and function of vector competence phenotypes, the biological and environmental factors that influence mosquito competence and then to apply this knowledge to mitigate mosquito-borne arboviral disease transmission.

The progress in understanding mosquito competence for arboviruses has provided the following conclusions: (1) There is a great amount of genetic variation within and between populations of mosquito vectors of arboviruses that likely extends to the genes controlling vector competence. (2) There is a great deal of phenotypic variation between individual mosquitoes and between populations of mosquitoes for every component of vector competence studied. (3) There are substantial numbers of different genes that influence every aspect of mosquito competence for arboviruses and the influence of these genes can differ depending on the mosquito population and the environmental conditions. (4) There are many different environmental factors that occur in both the mosquito larval and adult stages that influence every aspect of mosquito competence. (5) The effects of environmental factors on mosquito competence for arboviruses influence one another and interact with mosquito genetic factors in largely unpredictable and complex ways.

The last conclusion poses the greatest challenge in making progress on characterizing and understanding the causes of variation in mosquito competence for arboviruses. Variation in mosquito competence between individual mosquitoes and populations of mosquitoes is the result of the interplay between currently largely unknown genetic factors influencing mosquito competence and diverse complex environmental factors that are also largely unknown. The ability to translate DNA sequence and genomic information into effects on actual phenotypes is rudimentary and scientists are not very good at doing this [168,169]. Although there are now numerous candidate genes known to influence mosquito competence, the norms of reaction or how any one of these genes reacts under the array of environmental conditions available in Nature is unknown [169]. In addition the levels of polymorphism, the alleles and their distributions for any of the literally hundreds of candidate genes and the influence of allelic polymorphism of these genes on mosquito competence for arboviruses are also unknown.

The continuing identification of mosquito genes that influence vector competence for arboviruses will accelerate with the development and application of new developing technologies for gene identification. However it is essential that studies begin that will determine the norms of reaction of such genes, the population genetics of these genes, and identify the causes that determine the distribution of these genes in mosquito populations. What has been missing in the efforts to characterize mosquito competence for arboviruses using genomics has been an assessment of naturally occurring determinants, their variants, and how these determinants have evolved. Characterizing the evolution of vector competence is essential to understand the factors that shape genotype and phenotype distributions within a species and characterizing the factors that might influence vector competence in the future. For example, although it is likely that future changes in climate will influence vector competence and the evolution of mosquito-pathogen systems, there is not nearly enough information about these systems to predict specific changes in the phenotypes of mosquitoes such as how vector competence for arboviruses might evolve under anticipated climatic changes [170]. What are the types of information that are required to predict the transmission of any mosquito-borne pathogen under future conditions? Some of the required information has been outlined here that begins with identifying and characterizing genes controlling the traits associated with mosquito competence for arboviruses under realistic environmental conditions. Though a daunting challenge, this represents only part of the information that will be needed. Mosquito-borne arbovirus transmission is dependent on more than competence. The discussion of anticipating the effects of climate change on mosquito-borne disease illustrates the complexity of these very complex systems [170,171].

A key question is whether the genes responsible for vector competence are adaptive. This leads to several other important questions. What are the factors that have influenced the evolution of the genes controlling mosquito competence for arboviruses? What are the factors that now govern the distribution and frequencies of these genes in natural populations of mosquitoes?

There are many studies demonstrating that the presence of arboviruses in mosquitoes influence traits related to the fitness of the mosquito vector and these are reviewed elsewhere [172]. Although this might lead to the conclusion that mosquitoes have been under selection as a result of arboviral infections, there is little evidence proving mosquito evolution has been influenced by arboviruses. Similarly though there is speculation that there have been evolutionary consequences from malaria infection in *Anopheles* [170], there is little direct evidence for this. Though several arboviruses have been shown to influence fitness related traits or were pathologic in a variety of mosquito species [174,175,176,177,178,179,180,181], the impact of any of these fitness related traits or any pathologic effects that have been observed due to the arbovirus on mosquito evolution have not been demonstrated. Observing an effect of an arbovirus on a trait related to fitness is not proof positive that the effect is of any consequence, or proof that the trait is under natural selection due to the effects of the arbovirus on the mosquito. It is the influence of the specific trait on the total reproductive success of the organism that must be demonstrated. For example, effects on fitness that occur post reproduction will not be influenced by selection. Effects on fitness that occur in only a small portion of a mosquito population may not influence adaptation or have a net effect on reproductive success for a variety of reasons. 

Consider responses to selection for a trait for improved fitness when a mosquito is infected with an arbovirus but that this same selected trait results in low fitness in the absence of the arbovirus. Only a small proportion of a mosquito population generally encounters the virus even during large epidemics. Although genes will be selected because they increase fitness after exposure to the virus in the small portion of the population encountering the arbovirus, they may decrease in the great majority of mosquitoes not encountering the virus and more so in populations where the arbovirus is not present at all. For naturally occurring infections in mosquitoes, the minimum infection rate (MIR) generally recorded as the number of infected mosquitoes per 1,000, is normally <1 to 10 with rare instances being on the order of 50 for brief periods [182,183,184,185,186,187,188]. The overwhelming proportion of the mosquito population never encounter a virus during an epidemic and more often 100% of the mosquitoes in a population will not encounter an arbovirus during the long inter-epidemic periods when the arbovirus is not present or at low levels in the population. Hence most traits involved in vector competence were and/or are now likely under natural selection for other functions during the long periods in the absence of the arbovirus. It is likely these other functions have determined fitness differences and these are the functions that respond to selection pressures. It is difficult to imagine selection coefficients on competence traits high enough and persistent enough to have a major influence on mosquito evolution considering the small numbers of mosquitoes encountering the arbovirus and the long period when such selection would be relaxed in the arbovirus’ absence. The absence of any phylogenetic signature for mosquito capacity to transmit arboviruses is consistent with the hypothesis that genes controlling mosquito competence for arbovirus were not adaptations selected for competence [170]. In the majority of mosquito-arbovirus systems the closest phylogenetic relatives of the competent vector species are incapable of transmitting the particular arbovirus for a variety of different reasons. At the same time the vectors have more distant phylogenetic relatives that are capable transmitters. 

**Figure 3:**
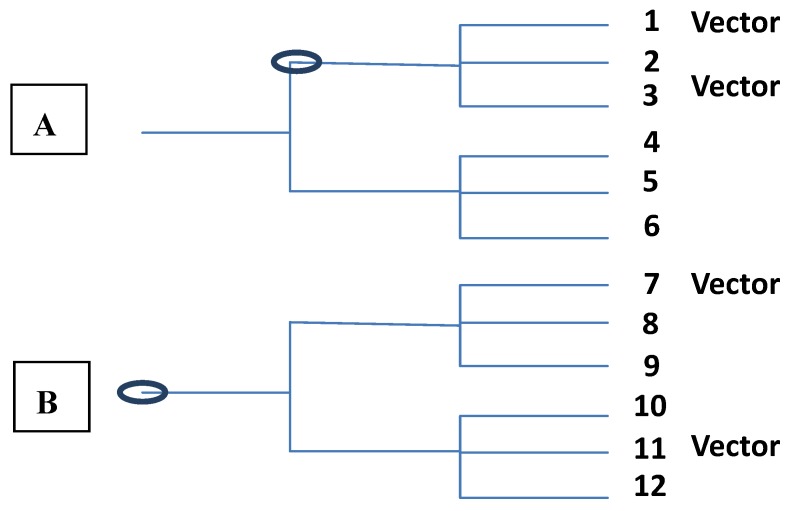
Phylogeny A is consistent with vector capacity being an ancestral trait from the common ancestor (circled). Hence it is common in the clade where sister species (1 and 3) are the vectors. Phylogeny B is consistent with either vector capacity being in the ancestor of both clades (circled) and being lost in the majority of descendant species, or that vector capacity is the result of convergent evolution in distantly related species (7 and 11).

The absence of phylogenetic signatures for vector competence is illustrated by the relationships of those *Culex* species that are capable vectors of WNV, those *Culicoides* species that are vectors of BTV, and the *Aedes* species that are vectors of both DENV and YFV [170]. For example, among the 217 or so species in the subgenus *Culex* only members of the *Cx. pipiens* complex, *Cx. univittatus* and *Cx. vishnui* are considered capable vectors of WNV. The nearest phylogenetic neighbors of these *Culex* vector species in this subgenus are not vectors. The same can be said for *Ae. aegypti* and *Ae. albopictus* the principal vectors of DENV and YFV. The primary *Culicoides* vectors of BTV are in disparate subgenera while species in the same subgenus as the vector species are themselves not vectors of BTV. The great majority of the species of *Stegomyia* here considered a subgenus, with the primary *Aedes* vectors of YFV, *Ae. aegypti*, *Ae. simpsoni *and *Ae. albopictus*, are not themselves vectors. Figure 3 illustrates two different phylogenetic patterns that may occur. One pattern suggests that the traits involved in vector competence were ancestral to the clade where the majority of the species are vectors. The second pattern suggests that either vector capability was ancestral to both clades and was lost in the majority of species, or that vector status was a consequence of convergent evolution in different clades.

The observations that sister members within the same clade of a vector species are not themselves vectors for the arbovirus, while species in different clades are vectors, makes it unlikely that vector capacity for arboviruses is an ancestral trait shared by the descendants of the common ancestor of the clade. The phylogenetic pattern that is most commonly found is more consistent with convergent evolution where mosquito species that perhaps share some common suite of traits for various reasons are more likely to be capable of transmission should they encounter a particular pathogen. This conclusion leads directly to the hypothesis that different mechanisms likely contribute to vector competence in disparate distantly related species. Tabachnick [170] believed that it is more likely that many traits that support vector ability including vector competence for arboviruses represent adaptations for completely different functions not related directly to competence. The vector competence traits are the effects of these adaptations and the adaptive function may still be under selection for this other function though not for the competence effect. Such traits are simply other effects of an adaptation [189]. There may be some traits that influence mosquito competence for arboviruses that were originally adaptations serving other functions and these may now be under natural selection when the mosquito encounters an arbovirus. These traits would be what Gould and Vrba [190] defined as exaptations. Though many mosquito traits involved in arbovirus transmission likely evolved due to other functions there are also likely some traits that may be ancestral in a particular group that include the aspects of the vector general body plan or bauplan that provide the basis or foundation for the evolution of vector capacity in the general sense [170]. For example, although only a few species of *Culex* transmit WNV, and only a few species of *Aedes* transmit YFV and DENV, the *Culex* bauplan has yet to provide a vector of DENV. The *Culex* bauplan, and the *Aedes* bauplan, *etc*. have differences that prevent the evolution of traits supporting competence for certain arboviruses. In general the *Anopheles* bauplan does not support the ability to vector arboviruses. It will be important to distinguish such circumstances, distinguish effects, exaptations adaptations, and just what are the different bauplans for vector competence phenotypes. What is needed is to characterize all of the mechanisms that influence vector competence and determine the other functions of the different traits and how they influence average reproductive success and are therefore subject to selection. Then it will be possible to determine those factors that shape the frequencies of these controlling genes in natural populations and determine the causes of the frequencies of competent mosquitoes in natural mosquito populations. 

## 8. Conclusions

There are environmental and genetic factors that influence variation in the ability of mosquitoes to transmit arboviruses. It is naïve to only address the influence of individual environmental factors on a trait such as competence for arboviruses without also considering interactions with genetic factors controlling competence. Environmental influences must be determined in the context of specific controlling genetic factors for specific traits that influence arbovirus epidemiology. The phenotype, and this includes all aspects of vector competence, is a function of genetic and environmental influences. However, the genotype and environment are simultaneously causes and effects since an organism is influenced by its genes, the environment also influences organisms, but the organism’s phenotype/genotype also influences the organism’s environment [168]. If there is anything we have learned since mosquito transmission of YFV was first demonstrated in Cuba at the start of the 20th century it is the complexity of the interactions of genes and environment, the complexity of nature and nurture. Mosquito competence for arboviruses is an excellent example of this complexity. There are many different traits that contribute to mosquito competence for arboviruses, each influenced by diverse genetic factors and a host of diverse environmental factors. It is apparent that there is great complexity in how environmental factors influence phenotypic variation in every component and trait comprising mosquito competence for arboviruses.

Different traits comprising mosquito competence for arboviruses are controlled by a variety of different yet still largely unknown genetic mechanisms and there is evidence suggesting that many different genes can influence the same trait. Different genes and very different genetic mechanisms may contribute to variation in a specific competence trait in different mosquito populations with the potential that different genetic mechanisms can produce the same competence phenotype in two different populations of the same species. Recall that different QTL were detected influencing susceptibility to infection for DENV in two different *Ae. aegypti* populations [78]. The norms of reaction for genotypes for competence traits have hardly been explored though it is now clear that norms of reaction of competence genotypes will be complex and that some will display nonlinear relationships between environmental variation for a factor, and adding to the complexity, the relationship between genotype and phenotypic expression across a range of variation for one environmental factor can be dependent on the variation in another environmental factor. Therefore even a complete understanding of genotypic variation controlling all of the mechanisms influencing vector competence will not provide the ability to predict resulting phenotypes unless there is a complete understanding of environmental influences on specific vector competence genotypes. Progress must entail understanding how environmental factors influence one another in their effect on the phenotype produced by specific genotypes. The answer to the issues facing medical entomology will not be provided simply by more genomics. Much more information is needed then can be provided by current genomics, sequencing and gene discovery studies. This will include work along the lines of what has been termed vector-borne disease system heterogeneities [191]. There is ample opportunity for new research projects focused on identifying and characterizing the complex heterogeneities in mosquito competence for arboviruses.

The importance of characterizing complex factors influencing mosquito competence is to be able to understand the epidemiology and causes of mosquito-borne diseases in order to be more effective in reducing their burden on human and animal health. The transmission parameters of vector-borne infections, *i.e*., those factors influencing the basic reproduction number (R_0_) of the pathogen, are influenced by many biological and environmental conditions that cannot be extrapolated to different situations [192] because of the reasons reviewed in this paper. The scope of the research that is required to understand the entire suite of factors encompassing vector-borne disease cycles previously described as the entire disease episystem [170] is daunting. Will it be essential to understand every factor and the influence of every factor on all other factors to be able to characterize the significance of vector competence variation? How accurate must our understanding of the details, the mechanisms, and the diversity of factors influencing mosquito competence for arboviruses have to be for sufficient understanding of mosquito-borne disease epidemiology? Are there environmental factors, genetic variants in controlling mechanisms, *etc*. that are slight enough so they can be ignored because they have little influence on biologically relevant variation? Science is at the very beginning of addressing these issues.

One of the greatest of the evolutionary biologists of the 20th century, T. Dobzhansky, believed that “nothing in biology makes sense except in the light of evolution” [193]. Mosquito competence for arboviruses is no exception. There is great potential to explore the evolutionary features of mosquito competence for arboviruses. This will require studies of complex issues that may not be as easily amenable to inquiry as the studies that focus exclusively on genomic approaches and gene discovery. Although identifying specific genes influencing competence for arboviruses will provide new opportunities to explore diverse genetic mechanisms, the challenge ahead will be to characterize the population genetic variation of the involved genes, assess the effects of genetic polymorphism on general physiology, behavior and ecology for example, and assess environmental factors, the norms of reaction, their evolution and determine the role of these genes in controlling phenotypic functions. These are difficult issues and they will require difficult approaches. It will require scientists willing to invest in risky and difficult undertakings. Do specific mosquito genotypes influence adaptive functions that influence arbovirus epidemiology? What is the significance of the functions of these genes? What is the variation? Why? The answers to these issues will require moving beyond gene discovery. Pursuing these issues will be the next hurdle for understanding vector-borne disease. This will be critical to develop new strategies to control the diseases caused by arboviruses including reducing competence in natural mosquito populations using genetic and environmental means and thereby providing another tool for humans to reduce the burden of mosquito-borne diseases.
